# Hoarding Disorder: A Case Report

**DOI:** 10.3389/fpsyt.2017.00112

**Published:** 2017-06-28

**Authors:** Daniela Vilaverde, Jorge Gonçalves, Pedro Morgado

**Affiliations:** ^1^Hospital de Braga, Braga, Portugal; ^2^Life and Health Sciences Research Institute (ICVS), School of Medicine, University of Minho, Braga, Portugal; ^3^ICVS-3Bs PT Government Associate Laboratory, Braga, Portugal

**Keywords:** hoarding disorder, obsessive–compulsive disorders, antipsychotics, psychotherapy, clinical case study

## Abstract

Hoarding disorder is characterized by a persistent difficulty discarding items, the desire to save items to avoid negative feelings associated with discarding them, significant accumulation of possessions that clutter active living areas and significant distress or impairment in areas of functioning. We present a case of a 52-year-old married man who was referred to the psychiatry department for collecting various objects that were deposited unorganized in the patient’s house. He reported to get anxious when someone else discarded some of these items. This behavior had started about 20 years earlier and it worsened with time. The garage, attic, and surroundings of his house were cluttered with these objects. On admission, in the mental status examination, it was observed that the patient was vigil, calm, and oriented; his mood was depressed; his speech was organized, logic, and coherent; and there were no psychotic symptoms. A psychotherapeutic plan was designed for the patient, including psychoeducation, cognitive restructuring, and exposure to discarding objects. A pharmacological treatment with fluvoxamine 100 mg tid and quetiapine 200 mg was added to the therapeutic plan, with the progressive improvement of the symptoms. Nine months later, the patient was able to sell/recycle most of the items. Studies evaluating treatment for HD are necessary to improve the quality of life of the patients and to reduce the hazards associated with the disorder.

## Introduction

The concept of hoarding was defined in 1996 as a behavioral phenomenon of acquisition of objects and failure to discard objects (1).

Compulsive hoarding was first included as diagnostic criteria for obsessive–compulsive personality disorder in the Diagnostic and Statistical Manual of Mental Disorders—third Edition Revised. Clinical hoarding began to be considered secondary to some disorders, such as dementia, residual schizophrenia, eating disorders, brain injury, autism spectrum disorders, or compulsive buying (2). Later, hoarding was considered a subtype or dimension of obsessive–compulsive disorder (OCD), with this behavior being seen in about 18–40% patients (2). However, hoarding would be seen frequently independent from other psychiatric disorders. After focusing on the epidemiological, phenomenological, and neurobiological aspects of hoarding, hoarding disorder (HD) was included as an isolated entity, in OCD spectrum, in the Diagnostic and Statistical Manual of Mental Disorders fifth Edition (DSM-V). The criteria for diagnosis of HD in DSM-V are a persistent difficulty discarding items, the desire to save items in order to avoid negative feelings associated with discarding them, significant accumulation of possessions that clutter active living areas, and significant distress or impairment in areas of functioning (Table 1) (3). Usually, these items are perceived to be useful in the future or to be esthetic or cause an emotional attachment (1, 4). If clutter is severe, it can cause threats to public health and safety (such as fire hazards and falls) (5).

**Table 1:** Diagnostic criteria for Hoarding disorder in the Diagnostic and Statistical Manual of Mental Disorders fifth edition (DSM-V).

A	Persistent difficulty discarding or parting with possessions, regardless of their actual value
B	This difficulty is due to a perceived need to save the items and to distress associated with discarding them
C	The difficulty discarding possessions results in the accumulation of possessions that congest and clutter active living areas and substantially compromises their intended use. If living areas are uncluttered, it is only because of the interventions of third parties (e.g., family members, cleaners, and authorities)
D	The hoarding causes clinically significant distress or impairment in social, occupational, or other important areas of functioning (including maintaining a safe environment for self and others)
E	The hoarding is not attributable to other medical conditions (e.g., brain injury, cerebrovascular disease, and Prader–Willi syndrome)
F	The hoarding is not better explained by the symptoms of other mental disorders (e.g., obsessions in obsessive–compulsive disorder, decreased energy in major depressive disorder, delusions in schizophrenia or another psychotic disorder, cognitive deficits in major neurocognitive disorder, and restricted interests in autism spectrum disorder)

In contrast to hoarding in OCD, the thoughts of not discarding in patients with HD are not considered by the patients to be intrusive, repetitive, or egodystonic (6). Besides, stress in HD is associated with the consequence of the clutter of the items and not related to the content of the thoughts associated with the reasons to not discard objects.

Compulsive hoarding affects about 2–5% of the population (7–9). Hoarding behaviors usually start at a subclinical level in early adolescence and worsens with each decade (10–12). Often the disorder becomes clinically significant only in middle-aged patients, with the distress associated to HD being caused by the intervention of others, such as relatives or local authorities (13). Stressful or traumatic events may be associated with the onset of hoarding symptoms (11).

Other psychiatric disorders often co-occur with HD. The most frequent comorbidity is a major depression, which can be present in up to 50% of the cases (14). Attention deficit/hyperactive disorder (ADHD) is also a common comorbidity with HD, with some studies suggesting association with inattentive symptoms of the disorder (15). Poorer general health is also associated with HD (16).

The cognitive behavioral model conceptualized for HD proposes that compulsive hoarding develops through emotional responses associated with the beliefs about possessions. This model proposes (1, 17):

1. Information processing deficits (including avoiding/delaying decisions with fear of making wrong decisions, self-report of inattentiveness and difficulty remembering/recording memories, and deficits in categorization/organization).
2. Emotional attachment and beliefs (of responsibility and control) for the possessions.
3. Maladaptive behavioral patterns (avoiding discarding objects which is associated with negative emotions, and acquiring and saving associated with positive emotions).
4. The cognitive behavioral treatment (CBT) designed to address HD involves assessment and psychoeducation on symptoms, motivation enhancement, cognitive restructuring, exposure to non-acquiring, and discarding and prevention of relapse (17, 18). One meta-analysis that focused on the results of CBT in patients with HD showed that symptoms decrease significantly after treatment with large effect size, especially with the difficulty to discard component of the disease (17). In the same study, rates of clinically significant changes were modest, with most patients continuing to score in the clinical range at posttreatment (17). Pharmacological treatment of HD disorder is not well established, since most studies evaluating treatment were made in patients with an OCD diagnosis with a hoarding component. Most of these studies used serotonin reuptake inhibitors, and the results were associated with poor response to treatment (19). A meta-analysis evaluated the effect of pharmacotherapy in patients with pathological hoarding (either diagnosed with OCD with hoarding component or HD) and response to treatment observed was 37–76% (20). In this study, only two studies focused on patients with HD. An open label trial used venlafaxine in monotherapy, in which 70% of the patients were classified as responders (decrease of ≥30% in the UCLA Hoarding Severity Scale and Saving Inventory—Revised and at least “much improved” on the Clinical Global Impression-Improvement) (21). The second study, a case series (*n* = 4) using methylphenidate, since there are some studies that point out the association between HD and ADHD, did not have significance in the resolution of hoarding symptoms (22).

## Case Report

We present a case of a 52-year-old married man, who has the sixth grade, and works as a mechanic. He has no previous history of medical conditions. As a psychiatric background, he reported depressive symptoms following the death of his only son 22 years ago.

**Figure 1:**
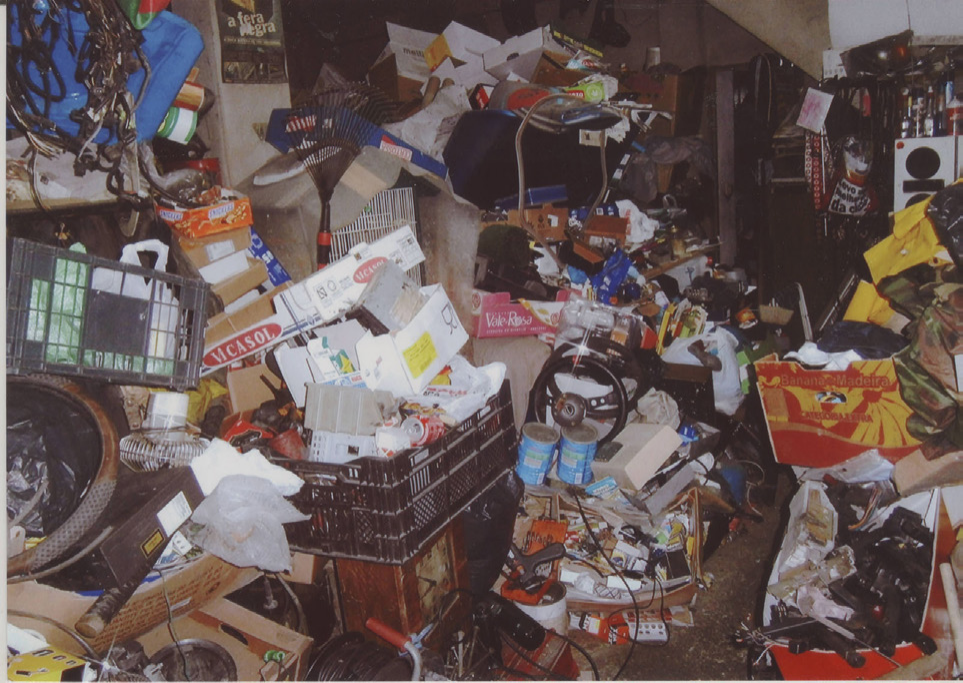
Patient’s house at beginning of medical intervention. Photo was taken and provided by patient. Consent for publication was obtained.

The patient was referred to treatment at the psychiatry department of Hospital de Braga for the first time by his family doctor for collecting various objects, predominantly stones, papers, and damaged pieces of cars that were deposited unorganized in the patient’s house. The patient collected these items because he thought they were valuable and/or usable in the future, but recognized that the collection was exaggerated. He could not discard any of these items, even if he never used them when needed. He reported to get anxious when someone else discarded some of these items. This behavior had started about 20 years earlier and it worsened with time. Despite, the thoughts of the possible use of these items were the reason why he could not throw out the possessions, these thoughts were not considered intrusive, repetitive, or egodystonic. The garage, attic, and surroundings of his house were cluttered with these objects (Figure 1). The clutter in his house is classified in to seven categories according to the clutter image rating (23, 24).

On admission, in the mental status examination, it was observed that the patient was vigil, calm, and oriented; his mood was depressed; his speech was organized, logic, and coherent; and there were no psychotic symptoms.

**Figure 2:**
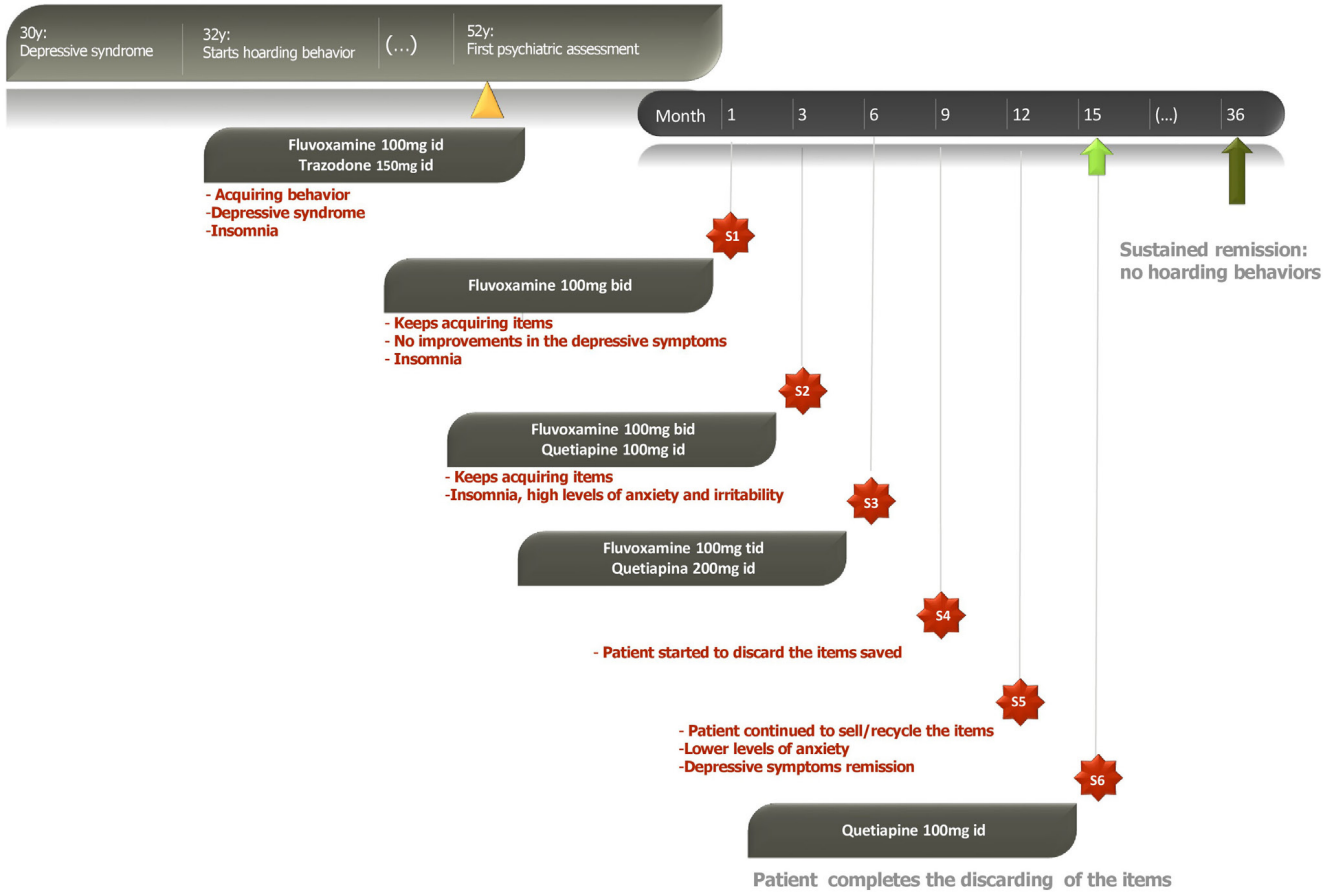
Timeline of the treatment plan.

**Figure 3:**
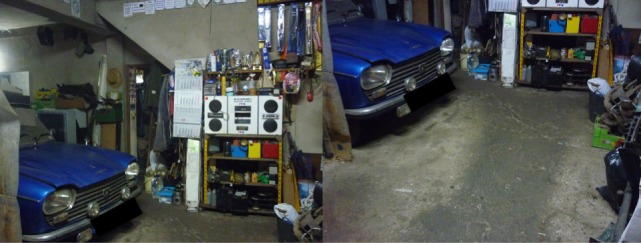
Patient’s house after medical treatment. Photo was taken and provided by the patient. Consent for publication was obtained.

A psychotherapeutic plan for the patient was designed. The goals were to understand and educate the patient on his symptoms and beliefs, so he could understand the need of treatment. Cognitive restructuring of the beliefs and exposure to non-acquire and discard the objects saved were also explored. Along with the psychotherapeutic plan, a pharmacological treatment with fluvoxamine 100 mg id and trazodone 150 mg id was made, to treat the coexistent depressive symptoms and insomnia. During the first 6 months of treatment, the patient continued to buy and collect objects. Then, fluvoxamine was gradually augmented to 100 mg tid and quetiapine 200 mg was added to the treatment plan, with progressive improvement in the symptoms and the patient being able to sell/recycle most of the items after 9 months of this treatment (Figure 2). The patient is currently medicated with quetiapine 100 mg id for insomnia and organized the spaces cluttered (Figure 3). The patient and his wife were very gratified with the results.

This study was performed in accordance with the provisions of the Declaration of Helsinki 2008 and was approved by the ethics eommittee of Hospital of Braga. We obtained informed written consent from the patient authorizing publication of clinical case and his photographs. His anonymity has been preserved.

## Discussion

We presented a patient who suffered from HD. The case was managed with both pharmacotherapy and a reduced number of psychotherapeutic sessions, which may be of interest in times of restrictions on available psychiatric and psychotherapeutic resources.

The clinical features of his object hoarding showed a typical pattern. The patient had an inability to discard objects and had an accumulation behavior with a long evolution over time. He was referred to the psychiatric treatment about 20 years after the beginning of the accumulation symptoms. Delay in seeking treatment for HD is very common and, if not treated, the evolution of the disorder occurs with progressive worsening of the behaviors.

The possessions collected did not have any value, and the thought that the possessions may be needed in the future did not have any characteristics of an obsession, such as being repetitive or egodystonic. In the same vein, the anxiety caused by the disorder was associated with the fact that he did not want to get rid of the items because he thought they could be valuable and no thought of something bad could happen, as typical in OCD. In addition, collected items disturbed the normal organization of his house and were cluttered.

Interestingly, the accumulation behavior began a few years after the death of his son. In fact, HD onset (as other obsessive-related disorders) is commonly associated with chronic stress and important life events (11, 25).

Hoarding disorder is mainly treated with CBT, including psychoeducation, motivational interviewing, classic cognitive techniques focused on dysfunctional beliefs, and exposures targeting sorting and discarding. Patients could also benefit with some pharmacological interventions.

In the presented case, the psychotherapeutic plan included a reduced number of sessions (basically due to limitations on available resources) and a combined pharmacologic approach.

Although no medications are currently marketed to treat HD, some studies identified benefits with the use of serotoninergic drugs in this disorder as in other disorders of the obsessive spectrum (19, 26). Treatment with fluvoxamine was chosen, not only for its characteristics of reduction of anxiety but also to address insomnia and depressive symptoms presented by the patient. Given that the patient did not tolerate trazodone, it was necessary to optimize the treatment for this problem, which constituted an opportunity for the use of quetiapine. Although there are no studies to evaluate the efficacy of quetiapine in this disorder, this case study illustrates its benefit that besides improving the sleep it seems to have contributed to lower the levels of anxiety in this patient.

Overall, the conjugation of described psychotherapeutic and pharmacotherapeutic approach contributed to significant improvement of the patient’s symptoms until sustained remission.

Future studies regarding the treatment of HD are necessary to address the difficulties of these patients and to improve their quality of life.

## Ethics Statement

This study was performed in accordance with the provisions of the Declaration of Helsinki 2008 and was approved by the ethics Committee of Hospital of Braga. We obtained informed written consent from the patient authorizing publication of clinical case and his photographs. His anonymity has been preserved.

## Author Contributions

DV and PM observed the patient, collected and analyzed the data. DV, JG, and PM wrote the paper. All authors approved the final work.

## Conflict of Interest Statement

The authors declare that the research was conducted in the absence of any commercial or financial relationships that could be construed as a potential conflict of interest.
